# Subsistence fishing patterns near food deserts

**DOI:** 10.1073/pnas.2519112122

**Published:** 2025-12-01

**Authors:** Savannah H. Swinea, Hailey Smith, Jonathan H. Grabowski, Sean P. Powers, Sara Wylie, Steven B. Scyphers

**Affiliations:** ^a^Department of Marine and Environmental Sciences, Northeastern University, Nahant, MA 01908; ^b^Stokes School of Marine and Environmental Sciences, University of South Alabama, Mobile, AL 36688; ^c^Dauphin Island Sea Lab, Dauphin Island, AL 36528; ^d^Department of Sociology and Anthropology and Social Science Environmental Health Research Institute, Northeastern University, Boston, MA 02120; ^e^Department of Sociology, Anthropology, and Social Work, University of South Alabama, Mobile, AL 36688

**Keywords:** subsistence, fisheries, blue space, social networks, policy

## Abstract

Fisheries are critical for sustaining waterfront communities. However, subsistence fishing is not well understood in the United States, despite its potential contributions to health and culture. We piloted a multivariable construct to classify subsistence vs. nonsubsistence fishers, identified the strongest predictor of participating in this practice, and tested for differences in place-based fishing motivations, behaviors, and community sharing. Among shore-based fishers in coastal Alabama, lower household income was the most powerful predictor of subsistence fishing. Subsistence fishers held more fishing motivations, targeted more specific fish groups, were more efficient in catching and keeping fish, and more frequently shared fish across social groups. Informed by these findings, we discussed management strategies to addressopportunities and barriers for shore-based subsistence fishing in coastal Alabama. More broadly, the framework piloted here offers a pathway to integrate subsistence fisheries into management using place-based evidence.

Fishing is important for livelihoods and food, contributing to sustainable development and food security (1). The role of subsistence fishing in reaching these goals has been overlooked, in part because measuring its prevalence has not been prioritized. To “subsist” is basal-level survival, but subsistence fishing reflects broader practices: exchanges among kinship groups; means to maintain social ties; and fishing for culture more than economics (2). Globally, subsistence fishing varies across peoples and places and occurs across sectors (commercial vs. recreational) and scales (large vs. small) (3). In the United States, subsistence fishing was recently defined in the Modernizing Recreational Fisheries Management Act (4), but only conceptually, offering no measurable approach to distinguish subsistence from nonsubsistence fishers. Without criteria for classification, subsistence fisheries remain neglected in US fisheries management and land planning, masking heterogeneity for those who fish for food.

Our objectives were to: 1) develop a construct to distinguish between subsistence and nonsubsistence fishers; 2) identify the most powerful predictor of being a subsistence fisher; and 3) evaluate how this status relates to fishing motivations, behaviors, and community sharing. We conducted a case study among people fishing from shore (shore-based fishers) to demonstrate the utility of this framework for managing subsistence fisheries. Shore-based fishing relies on access to green/blue space, yet shore-based fishers face challenges from limited mobility and access to fishing sites. Despite evidence that shore-based fishers differ behaviorally and demographically from boat fishers (5), these disparities are largely unaddressed in US management (6).

Our case study centers on Alabama’s coast, located in the US Gulf of America (GOA), also known as the Gulf of Mexico (Fig. 1*A*). Alabama has maintained the highest proportion of shore-based trips among GOA states for the past decade (7). Regionally, the GOA has among the highest recreational harvest rates in the United States, and GOA communities are disproportionately motivated to fish for food and eat more seafood than residents of other regions (8).

**Fig. 1.**
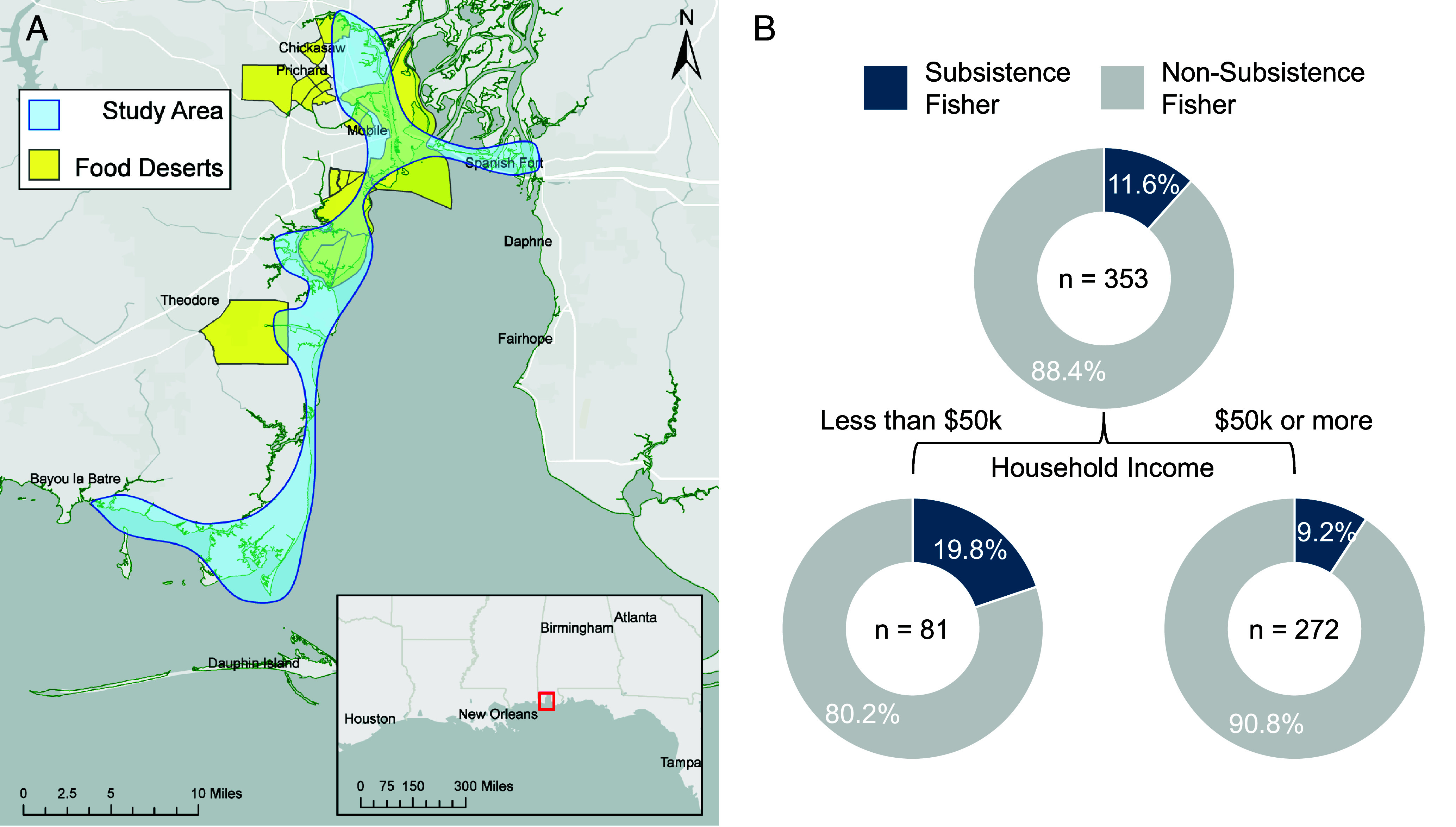
(*A*) Public fishing site sample frame and food desert census tracts in coastal Alabama (9). (*B*) Classification tree identifying the most powerful explanatory variable for being a subsistence fisher. Branches indicate divisions at α = 0.05. Value centered in each circle represents the number of individuals.

## Results

Using our construct (*Materials and Methods*), 11.6% of respondents (N = 41) were classified as subsistence fishers. Among several behavioral and demographic predictors, lower household income (less than $50 k) was the most powerful predictor of being a subsistence fisher (Fig. 1*B*; *P* = 0.046).

**Fig. 2.**
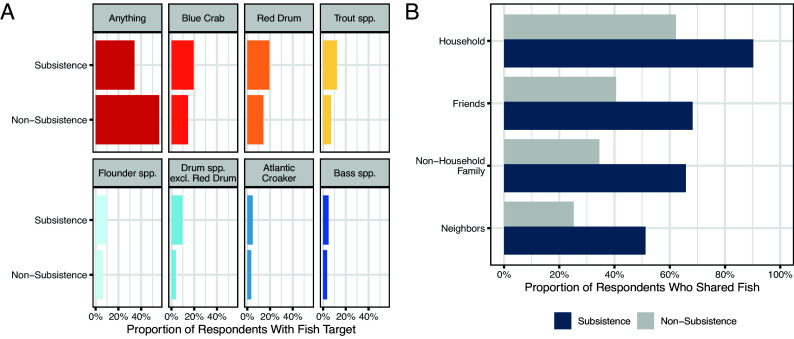
Differences in the proportion of subsistence vs. nonsubsistence fishers who (*A*) targeted fish groups and (*B*) shared fish with social groups.

Subsistence and nonsubsistence fishers diverged significantly in their place-based motivations, behaviors, and community sharing. For fishing motivations, subsistence fishers reported 10 motivations on average, where nonsubsistence fishers reported three (*P* < 0.001). The three largest discrepancies in motivations between subsistence and nonsubsistence fishers (followed by the difference) were safety (63%), therapy (52%), and fish diversity/abundance (46%); however, solitary vs. social fishing were similar across these groups. For target species, more subsistence fishers targeted specific fish groups including all top five targets, where nonsubsistence fishers more often reported no specific target (Fig. 2*A*; *P* = 0.015). For fishing outcomes, average catch per unit effort for fish kept was significantly higher for subsistence fishers (3.14 fish/hour) than nonsubsistence fishers (1.40 fish/hour), despite similar years fished and avidity. For community sharing, more subsistence fishers shared with all social groups (household members, nonhousehold family, friends, and neighbors) compared to nonsubsistence fishers (Fig. 2*B*; *P* = 0.001).

## Discussion

We piloted a construct to classify subsistence fishers and show how they differed from nonsubsistence fishers. Both predictors and outcomes can inform evidence-based policy. In coastal Alabama, low-income fishers were more often subsistence fishers; income-based policies from other states could be applied to support subsistence fishing. Subsistence fishers’ specific targeting and catch rates highlighted how changes in bag limits or other regulations could impact food procurement. Because subsistence fishing differs across contexts, reproducing our analyses elsewhere would yield different insights and policies. Existing subsistence policies sometimes rely on income-driven measures, while others incorporate residence time, location, and tribal status (10). Thus, diversity across subsistence fisheries precludes a single management strategy. We present a framework capable of transcending sectors that still results in place-based evidence for local policy.

Shore-based, public sites provide an indispensable avenue to access blue/green spaces (11). Alabama shore-based subsistence fishers held more place-based fishing motivations than nonsubsistence fishers that diverged most for safety, therapy, and fish diversity/abundance. Solitary vs. social fishing was similar for these groups, suggesting factors unrelated to companionship drove differences in safety motivation. Shore-based subsistence fishers, who may lack alternatives outside of public sites to fish for food, may face barriers that are biophysical (e.g., degraded habitats, stock healths), social (e.g., safety), or institutional (e.g., land privatization). With the majority of Alabama’s shorelines being privately owned, sustaining and introducing shore-based sites has been a priority of the state (12). Land planning must balance resource use, ecological integrity, and social belonging.

Effective policies must address opportunities and barriers for subsistence fisheries. For example, the contribution of subsistence fishing to food security must be weighed against local consumption trade-offs (nutrition vs. contamination concerns). In coastal Alabama, more subsistence fishers shared their catch with all social groups than nonsubsistence fishers. Notably, 39% of sampled public fishing sites were in food deserts, compared to 18% of census tracts (9; Fig. 1*A*), suggesting that subsistence fishing could presently, or ultimately, play a role in food security. Nutrient/contaminant profiles in local fish coupled with consumption and sharing behaviors would clarify this role.

The broader recognition of subsistence fishing in US legislation (4) was a decisive step for understanding this practice. However, subsistence fisheries research and management remain fragmented (13). The framework piloted here could be integrated with fisheries survey programs to characterize US subsistence fisheries while recognizing nuances, opportunities, and barriers inherent to these diverse fisheries. Implementing this framework would unify research behind subsistence fisheries, allowing evidence-based policies or management strategies to emerge. In our case study, we described fisheries management and land planning strategies to sustain the shore-based subsistence fishers of coastal Alabama, and the development of place-based strategies for other subsistence fisheries could emulate this approach. To realize the total capacity for fisheries to enhance sustainability and food security, we must document subsistence fishing as a pathway for providing fish for food among American communities.

## Materials and Methods

We deployed surveys at public, shore-based fishing sites along Mobile Bay, Alabama, from May 2023 to May 2024 (Fig. 1*A*; N = 355). Human subjects research was approved by the University of South Alabama’s IRB (Protocol #23-176), and informed consent was obtained on-site. The survey addressed subsistence characteristics, fishing motivations and behaviors, community sharing, and demographics. We classified fishers as “subsistence” if they met three criteria: 1) reported subsistence as a fishing motivation that day; 2) had eaten any self-caught local fish that month; and 3) had brought home fish for themselves or others to eat. This construct measured motivational and behavioral dimensions across day, month, and lifetime, allowing us to disaggregate these groups with greater nuance than before (13, 14).

To evaluate predictors of being a subsistence fisher, we applied tree-based classification models using the Chi-squared Automatic Interaction Detection Method (CHAID) with predictors from three categories: fishing behavior (general frequency of recreational fishing, days fished in bays/rivers, nearshore, or offshore in preceding 12 mo, fishing avidity, years fished); social behavior (frequency of selling/trading fish, frequency of fishing alone, with family, or with friends); and demographics (household income, gender, year born, race/ethnicity, highest level of education, financial assistance status). In testing across subsistence and nonsubsistence fishers, we applied the Wilcoxon signed rank test to: place-based fishing motivations; solitary/social fishing behaviors; catch per unit effort for fish kept (number of fish caught and kept divided by time spent fishing); years fished; and avidity. We applied the PERMANOVA test to fish community targeted that day; and community of social groups with which fishers shared their catch.

## Supplementary Material

Appendix 01 (PDF)

## Data Availability

Survey data and code data have been deposited in GitHub (Subsistence Fishing: https://github.com/savannahswinea/SubsistenceFishing) (15).
