# Supplementary material for: Subsistence fishing patterns near food deserts
Source: Proc Natl Acad Sci U S A. 2025 Dec 1;122(49):e2519112122. doi: 10.1073/pnas.2519112122 (PMC12704766; doi:10.1073/pnas.2519112122)
Supplement: Supplementary file 1 — Appendix 01 (PDF) [file pnas.2519112122.sapp.pdf]

PNAS

Supporting Information for
Subsistence fishing patterns near food deserts

Savannah H. Swinea, Hailey Smith, Jonathan G. Grabowski, Sean P. Powers, Sara Wylie, and Steven B. Scyphers

Savannah H. Swinea
Email: swinea.s@northeastern.edu

This PDF file includes:

Supporting text

Supporting Information Text
Survey Instrument.

1. Target Species today: Anything Other: ____
2. Catch today (#, species, kept or released): ____
3. Minutes gear in the water: ________________
4. Which of these words describe why you fish at this site? (Check all that apply)

☐ Aesthetics/Scenery ☐ Access ☐ Community/Sense of Belonging ☐ Convenience ☐ Culture ☐ Happy/Proud Feelings ☐ Fish Diversity/Abundance ☐ Healthy/Clean Environment ☐ Historic ☐ Infrastructure (Piers, Seawalls) ☐ Parking ☐ Past Experiences or Milestones ☐ Safety ☐ Spiritual ☐ Subsistence ☐ Therapeutic ☐ Wilderness ☐ Other: ___________

5. How often do you use or access your coast for the following recreational activities?

	Never	Less than once per year	Yearly	Monthly	Weekly	Daily
Recreational Fishing	☐	☐	☐	☐	☐	☐

6. Which of the following statements best describes you?

☐ Fishing is an enjoyable, but infrequent activity that is incidental to other travel and outdoor interests. I am not highly skilled in fishing, rarely read fishing articles, and do not own much fishing equipment beyond the basic necessities.
☐ Fishing is an important, but not exclusive outdoor activity. I occasionally read fishing articles and purchase additional equipment to aid in fishing, my participation in fishing is inconsistent, and I am moderately skilled in fishing.
☐ Fishing is my primary outdoor activity. I purchase ever-increasing amounts of equipment to aid in fishing, go fishing every chance that I get, consider myself to be highly skilled in fishing, and frequently read fishing articles.

7. How many years have you been fishing? __________
8. Who do you fish with and how often? ____
9. Days fished in the last year: ________Bays and Rivers __________Nearshore __________Offshore
10. Do you ever bring home fish for you or others to eat? Y / N
11. How many meals with fish do you consume in a month? ________
12. How many meals are caught locally? _________
13. Who do you share your catch with? Household members Non-household family and relatives Neighbors Friends

14. How often do you sell and/or trade your catch for other goods? Never Rarely Sometimes Often Always

15. What year were you born? ______________________

16. What is your gender? Male Female Other

17. What is your highest level of education? Less than high school High school diploma or GED Some college or 2 year degree Bachelor's degree Master's degree Law or MD Doctorate (PhD)

18. What is your race or ethnicity? White Black or African American American Indian or Alaska Native Asian Native Hawaiian or other Pacific Islander Hispanic or Latino Other: ______________

19. What was your total household income in 2022? $15k or less $15,001 to 25k $25,001 to $35k $35,001 to $50k $50,001 to $75k $75,001 to $100k $100,001 to $150k $150,001 to $250k More than $250k

20. Are you on any form of financial assistance? Y / N
